# Patterns in the distribution of Ecuadorian amphibian type localities

**DOI:** 10.1038/s41598-026-53084-4

**Published:** 2026-05-14

**Authors:** Diana Székely, Diego Armijos-Ojeda, Paul Székely

**Affiliations:** 1 Museo de Zoología, Universidad Técnica Particular de Loja, San Cayetano Alto, calle París s/n, 110107 Loja, Ecuador; 2 Faculty of Natural and Agricultural Sciences, Ovidius University Constanța, Al. Universității no.1, 900470 Constanța, Romania; 3 Laboratorio de Ecología Tropical y Servicios Ecosistémicos (EcoSs-Lab), Departamento de Ciencias Biológicas y Agropecuarias, Facultad de Ciencias Exactas y Naturales, Universidad Técnica Particular de Loja, San Cayetano Alto s/n, 110107 Loja, Ecuador

**Keywords:** Conservation policies, Conservation priority, Endemic fauna, *Loci classici*, Spatial analysis, Ecology, Ecology, Zoology

## Abstract

In taxonomy, the type locality of a species is a fundamental concept, as relevant to the species description as the definition of morphological characters. Although they hold a high intrinsic scientific, cultural and social value, their conservation has been globally overlooked. As a showcase, we focused our study on a megadiverse country (Ecuador) and a diverse group of vertebrates (amphibians). We georeferenced the type localities of all amphibian species reported from Ecuador, evaluated chronological changes in their distribution, assessed the factors that affect their location, and emphasized areas of high conservation value. We found that the distribution of type localities is affected by road access and closeness to inhabited centers, most are unprotected, and more intense sampling in an area leads to a higher number of type localities there, while the relationship with species richness is more complex. Our study reveals “hotspots” of type localities, and in many cases their protection status can be improved through marginal extension of already established reserves. These sites represent a priority for the conservation of microendemic species (over 20%), and they have potential to bolster conservation actions given the social interest in their historical dimension and attractiveness for the general public.

**Supplementary Information:**

The online version contains supplementary material available at 10.1038/s41598-026-53084-4.

## Introduction

Ecuador, the smallest Andean country (1% of the area of the South American continent), is a hotspot for biodiversity^1^ and has one of the largest numbers of amphibian species in the world^2,3^, with a mixture of wide-ranging species mostly characteristic to Amazonian fauna, along with endemic species found nowhere else^4^. Relative to its size, it is the world’s most diverse country, with roughly 2.3 species per 1000 square kilometers, 69 times higher than that of the United States’, 17 times higher than Brazil’s, 11 times higher than Mexico’s, 5 times higher than Peru’s, and 3 times higher than Colombia’s^5^. Documenting its diversity is an ongoing scientific effort; although this endeavor started in the middle of the 19th century, a staggering 12% new amphibian species have been added in the last 5 years alone. The poorly known status of amphibians is a global pattern, amphibians showing one of the highest recent rates of new species description among the terrestrial vertebrates^3,6^. Amphibians are also the most threatened among terrestrial vertebrates, with a worrying trend of increased risk of extinction for newly discovered species, due to restricted ranges, small population sizes and impact from human activities^7^.

The first step in quantifying biodiversity is species delimitation. This starts with the formal description of species, by permanently attaching a Linnaean name to an organism (a tangible physical material^8^), assigning it as primary type (optimally a holotype). This type material serves as a reference point against which all future comparisons are made. Intrinsically associated with the primary type specimen is its locality of provenance – the type locality^9^. Detailed information regarding the geographical location where the type material was collected is as important as the clear description of morphological characters^10^.

Type localities are not necessarily representative of the range of the species, as they depend on logistical access, and they likely reflect the distribution of collectors and their interests^11^. However, in the case of species with restricted ranges, the locality is representative of the special conditions necessary for their survival^12^; this can be especially relevant in the case of land vertebrates with low vagility such as amphibians. Type localities possess taxonomic value, representing the site where the species can be found; they offer the possibility of obtaining additional data in the future. However, *loci classici* also bear historical relevance, having been visited by important personalities such as naturalists and herpetologists, holding thus cultural heritage^13^.

In the context of the current unprecedented human mediated extinction, conservation strategies are focused mostly on iconic species and highly biodiverse sites, as close to pristine as possible^14,15^. Because the funding is limited and nature conservation needs to be prioritized, type localities have been disregarded when considering the establishment of protected areas, although they are likely to suffer degradation or even rampant destruction due to their inherent accessibility and closeness to inhabited centers. An improved understanding of the spatial distribution of the type localities is a first step for identifying their needs^16^.

In this context, our main objective was to assemble the information available on type localities for all amphibian species recorded from Ecuador. Based on a systematic compilation of type localities for all amphibian species that are known to occur in Ecuador, we provide a quantitative overview of the geographical distribution, evaluate chronological changes, and pinpoint factors that impact their location. We reveal “hotspots of type localities” – areas from which a high number of species/ high proportion of microendemics were described, and assess their current state and potential for conservation.

## Methods

### Dataset

The reference point for the list of amphibian species that are reported from Ecuador was Bioweb – Anfibios del Ecuador (last accessed august 2025^17^). The taxonomy was updated according to Frost^2^; we included only currently valid species, and omitted synonyms. Information on primary type specimen and locality was based on the original publication, corrected by additional sources such as taxonomic monographs, online museum databases and published type catalogues as needed. Because errors occur and paratypes sometimes prove to belong to different species compared to the holotype^18–20^, we chose to focus only on the primary type (holotype whenever available, and if not, on syntypes, lectotypes and neotypes), and considered secondary type material (paratypes, allotypes, other reviewed material for species description) as beyond the scope of this work. When the location for neotype improved the detail of the original description, this location was used.

We generated a revised geo-referenced list of type localities of the amphibian species present in Ecuador. One species - *Aquarana catesbeiana* (Shaw, 1802) - is exotic and was excluded from the dataset; the only amphibians that occur on the Galapagos islands are introduced^21^, so we restricted our analysis to continental Ecuador. For each species, the geographical location was retrieved from the original text. Whenever possible, for ambiguous localities, we cross-referenced the description of the locality given in the original paper with collector itineraries, museum catalogues, or old maps. The species’ known geographic range was taken into account only to disregard localities that are in obvious error (e.g., for improbable trans-Andean distribution). Geographical data varied in their detail: while most recent publications offered precise locations (including high resolution GPS coordinates), some older references gave wide areas and general hints (e.g., America, Ecuadorian Andes, “probably from Brazil”), or even incorrect ones (e.g., Virginia).

When georeferencing, we included a field in the database, based on expert opinion, which provides metadata-type information on the geographical accuracy, using a ranked scale from 0 to 5^13^. Within this scale, an accuracy level equal to 0 implies that the geographical coordinates are expected to be very accurate and located within a circular area of expected error with a diameter of < 500 m (266 type localities). Level 1 implies 0.5–1 km (299 type localities), level 2 implies 1–5 km (52 type localities), level 3 implies 5–10 km (12 type localities), while level 5 was assigned to localities which were impossible to determine (e.g. western Andes, Ecuador, Pastaza river), or considered incorrect because species has a divergent distribution (western Andes locality given to an Amazonian species). The type localities with low geographic accuracy (estimated over 5 km radius or impossible to assign – 66 records) were discarded from geographical analysis.

### Data analysis

Statistical analyses were carried out in R software vers. 4.3.2^22^, using packages ggplot and tmap^23^ for graphical representation, while spatial analysis and maps were produced in QGIS software version 3.40 Bratislava.

The full dataset (type localities from both inside and outside Ecuador) was used for general plotting of all species with accurate location (*n* = 629). We evaluated how the distance between type locality and the centroid of species distribution is associated with the year of species description through a Spearman correlation, using all species with available data (precise type locality and range map; see below).

For the rest of analysis, we focused on the amphibian type localities that lie within Ecuador (*n* = 505). We detail the spatial distribution of type localities by region, province, type of ecosystem (classification as given by Ron et al.^24^). We evaluated how many are located inside the Ecuadorian National Network of Protected Areas (Sistema Nacional de Áreas Protegidas - SNAP) and the overlap between type localities and provincial administrative boundaries and land use cover (http://ide.ambiente.gob.ec/^25^). Elevation data was extracted for each data point (type locality) using the complement “point sampling tool” of QGIS, from elevation digital model (https://dataspace.copernicus.eu/explore-data/data-collections/copernicus-contributing-missions/collections-description/COP-DEM) with a resolution of 90 m. We tested for association between interval of species description (before 1950, 1950–2000, after 2000) and type locality elevation (< 1000 m a.s.l., 1000–3500, > 3500 m a.s.l.) by means of a chi-square test, and checked for a relation between year of description and the elevation of the type locality using a Spearman correlation.

For the evaluation of type locality densities, we created a 10 × 10 km grid over the surface of continental Ecuador. To check for patterns in the geographic distribution of type localities, we evaluated the relationship with species richness, sampling intensity, and logistic bias (cities and roads). As a proxy for species richness, we downloaded the range polygons from IUCN Red List^26^ for all amphibians reported from Ecuador, filtering for extant and possibly extinct, native, resident species. This produced range maps for 601 species. We added range maps for an additional 86 species, by searching for occurrence points in primary literature, using Google Scholar, and specific searches in scientific journals dedicated to publishing range extensions (Herpetological Review, Check List, Mesoamerican Herpetology); we added field data available at Museo de Zoología, Universidad Técnica Particular de Loja (MUTPL). Based on these records, we developed extent of occurrence maps using Minimum Convex Polygons (MCP) for species with at least 3 occurrence points (45 species)^27^, by creating 1-km radius buffers around occurrence points for those with one locality (25 species), and a line uniting the two localities with a buffer of 1 km around for those with 2 localities (17 species). Localities were defined as occurrence points located at least 1 km apart. Although MCP tends to overestimate the extent of occurrence of a species, we considered it an adequate proxy of the geographical range of the species, taking into account the restricted range and the small amount of information available for most of the Ecuadorian amphibians that had no IUCN range maps^28^. We overlayed species ranges to calculate species richness as the sum of species cooccurring over the 10 × 10 km grid. We modeled the relationship between the number of type localities and species richness per grid cell. Since the model with a Poisson distribution showed overdispersion, we used a zero-inflated model (zeroinfl function of the pscl package) with a negative binomial distribution (with log-link), which models separately the count and the zero component^29^.

Additionally, based on range maps, we arbitrarily assigned as microendemics the species that have been reported from only one locality, those that were reported from two localities separated by less than 50 km in a straight line, and those with an extent of occurrence < 100 km^2^, which partially fits the IUCN B1 criterion of Critically Endangered species.

As a measure of sampling intensity, we performed a search for amphibian records from GBIF using the rgbif package (search parameters: Basis of record - Material sample, Material citation, Preserved specimen; Country - Ecuador; Has coordinates - True; Has geospatial issues - False; Occurrence status - Present) on 26 July 2025 (10.15468/dl.ghp73k). The downloaded records (*n* = 150,956) were restricted to data with a coordinate uncertainty < 5 km (*n* = 131,968), cleaned using package CoordinateCleaner by excluding problematic records (equal lat/lon *n* = 2, zero coordinates *n* = 32, country capital *n* = 960, country centroid *n* = 15), records falling outside Ecuador (*n* = 1884), those with individual count = 0 (*n* = 20), and those older than 1945 which we considered could have low coordinate precision (*n* = 9,552), for a total of 119,505 retained records. Afterwards, we merged records that were identical in terms of location coordinates, date and institution. This resulted in a “total sampling occasions” dataset (*n* = 25,309), which we used as a measure of sampling intensity. We modeled the relationship between the number of type localities and the number of samplings per 10 km cell in a grid over continental Ecuador. Similarly to species richness, the model with a Poisson distribution showed overdispersion, so we used a zero-inflated model with a negative binomial distribution.

We evaluated the influence of logistic accessibility on the spatial distribution of type localities and also sampling effort^30^, using the *sampbias* package^31^, which quantifies the impact of specified geographic features through a Bayesian analysis of sampling rates as a function of distance from biasing factors. As potential factors we used cities and roads (downloaded from https://www.geoportaligm.gob.ec), setting the resolution for analysis at 1 km, and used continental Ecuador as a layer mask.

## Results

**Fig. 1:**
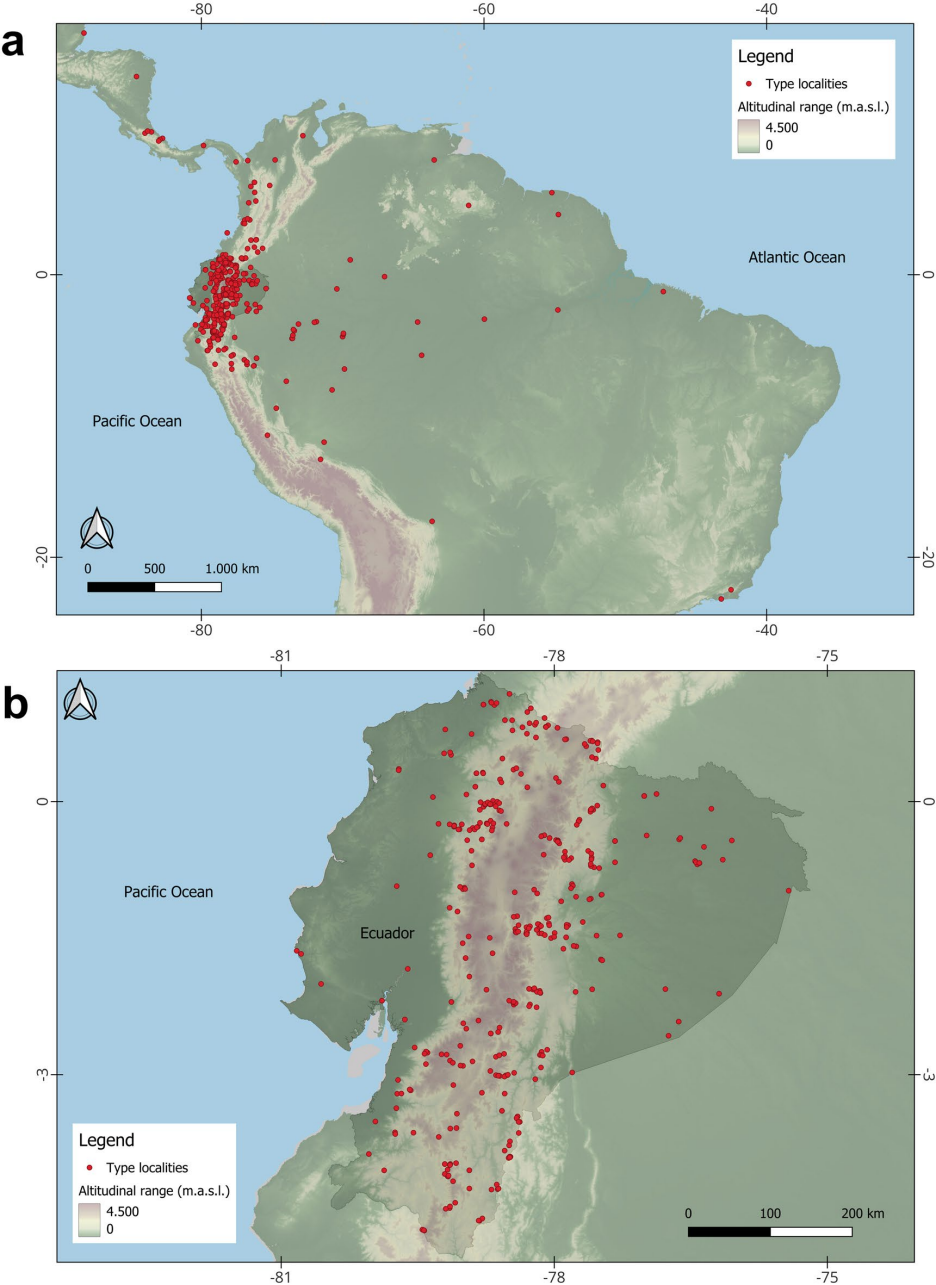
Type localities of amphibian species that have been reported from Ecuador: (**a**) all species reported from Ecuador, (**b**) type localities from Ecuador only.

Our dataset consisted of 695 amphibian species (Fig. 1a), of which 534 (76.8%) have their main type material originating from Ecuador (Fig. 1b) and 326 (46.9%) are endemic to the country. An additional 79 amphibian species that have their type locality in Ecuador have been synonymized and were not included in the dataset. Of the 63 genera that are represented in the country, 18 have the type species described from Ecuador. Over one in five (151 of the 695 recognized species present in Ecuador, i.e. 21.7%) are microendemic, of which 69 are known only from their type locality and vicinities. The first amphibian species to be described are amply distributed and their type material comes from various countries in Latin America, such as Brazil, Surinam, Peru or Costa Rica; however, this situation rapidly changed, and, after 1850, the species that have their type locality in Ecuador are predominant (Supplementary Fig. S1a). The distance between the type locality and the centroid of the species distribution shows a reduction over time (*ρ* = -0.585, *p* < 0.001).

### Geographical analysis of Ecuadorian type localities

**Fig. 2:**
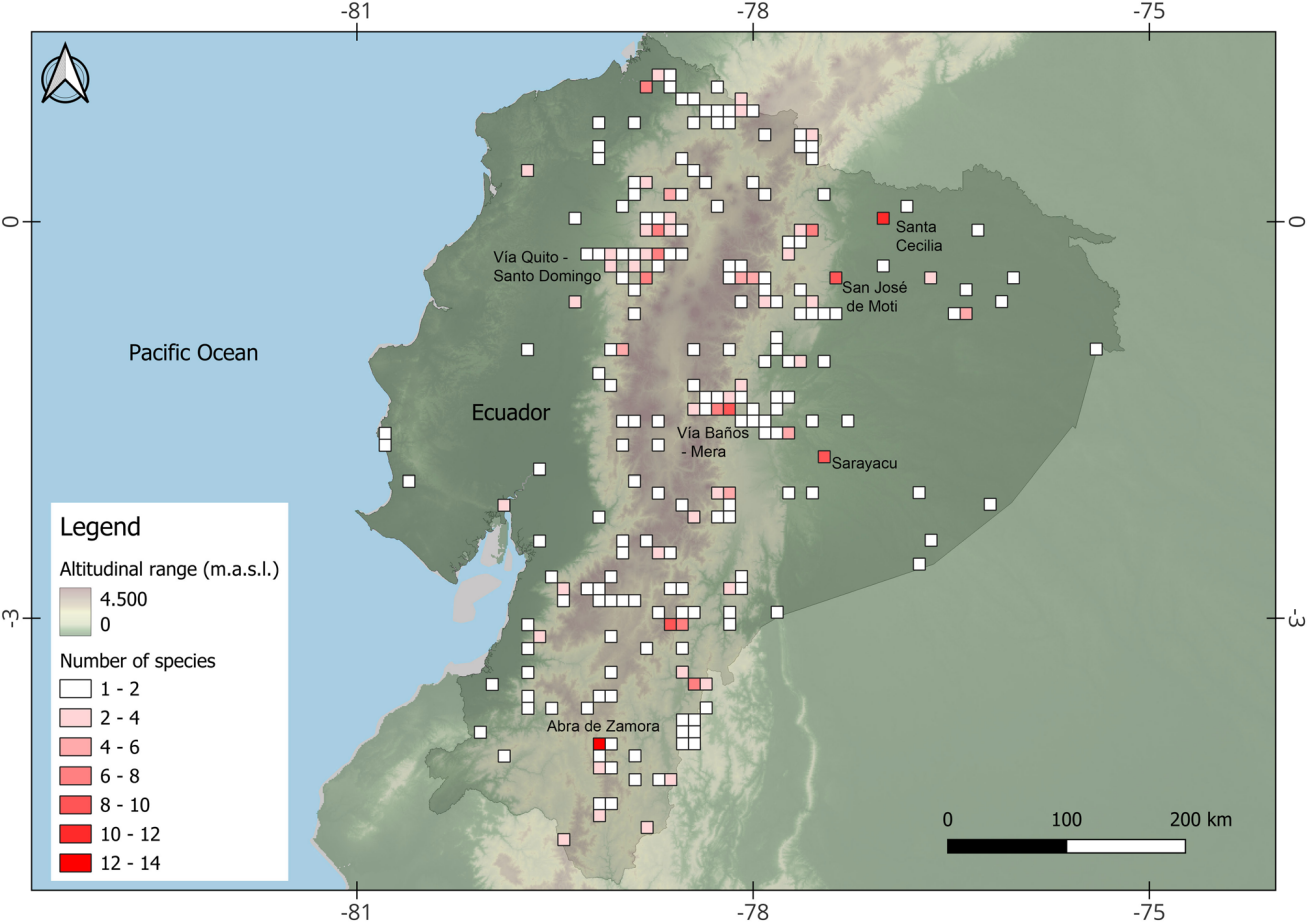
Density of type localities of amphibian species described from Ecuador, over a 10 × 10 km grid. Important clusters of type localities are indicated by name.

For 66 of the total species, type locality was too vague, or no type locality was cited in the original publication. Specifically, for 28 species described from Ecuador, type locality could not be determined with sufficient accuracy (authors mention Ecuador, Western Ecuador, Ecuadorian Andes, Andes of western Ecuador, Pastaza Valley, Pastaza River, Valley of Quito, mountains above Chimbo as the location). For the rest (*n* = 505 species), their type localities have a patchy distribution (Fig. 2), with concentration points in Abra de Zamora and the city of Loja (14 type localities in one grid cell, of which 57.1% microendemic species), Santa Cecilia (11 type localities over one grid cell, none microendemic), road between San Vicente and Plan de Milagro (18 type localities over 4 grid cells, 27.8% microendemics), road between Baños and Mera (29 type localities over 8 grid cells, 55.2% microendemics), Sarayacu (10 type localities over one grid, no microendemics), San José de Moti (9 type localities over one grid, no microendemics) and the old road between Quito and Santo Domingo de los Colorados (21 type localities over 6 grid cells, 9.5% microendemics).

**Fig. 3:**
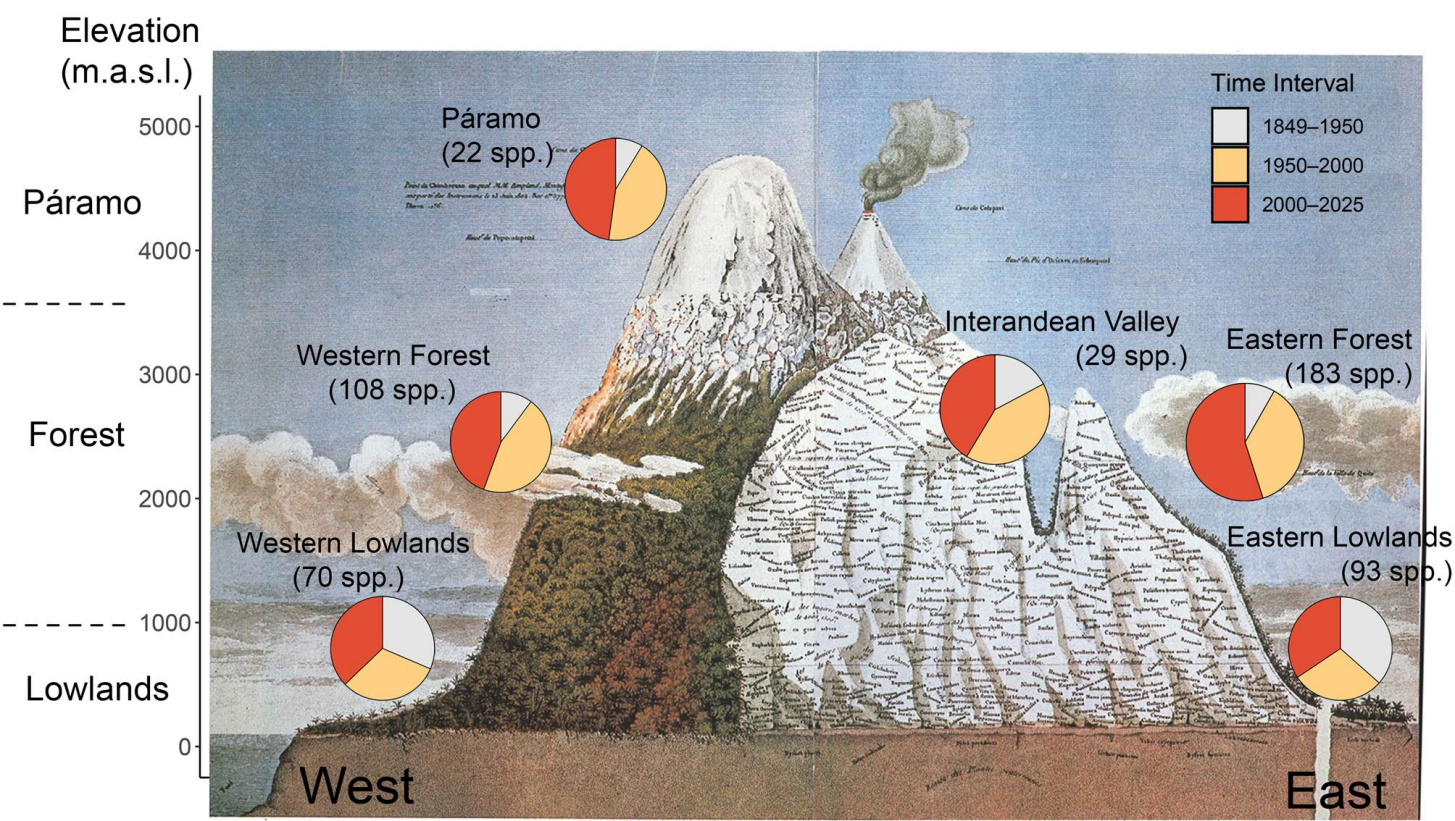
Distribution of type localities according to their altitude, and proportion of species description according to the time interval of their description. Background showing Alexander von Humboldt’s Andes profile from *Tableau Physique.*

The province with the highest number of type localities is Morona Santiago (65 species), followed by Napo (52), Pichincha (45) and Pastaza (43). The distribution of type localities by ecosystem shows that most are located in the Eastern Montane Forest (175), the Western Montane Forest (105), and the Amazonian Tropical Forest (77) ecosystems; intermediate numbers are found in Choco Tropical Rainforests (25), Eastern (24) and Western Foothill Forests (33), as well as Andean Shrub (29) and the Páramo (22); the fewest type localities are present in dry habitats, i.e. the Western Deciduous Forest (13) and the Dry Shrub (2). In terms of elevation, there is a weak positive trend of species with type localities from lower altitudes being described sooner (Spearman correlation, *ρ* = 0.26, *p* < 0.001). There are significant differences in terms of the time interval when the species were described (*χ*^2^_10_ = 51.693, *p* < 0.001) as a function of the ecosystem of the type locality; we notice that a higher proportion of the species that have their type localities in the lowlands were described in historical times (i.e. before 1950), while there is an intensification of species described from Páramo and Eastern Forest in recent years (after 2000; Fig. 3).

**Fig. 4:**
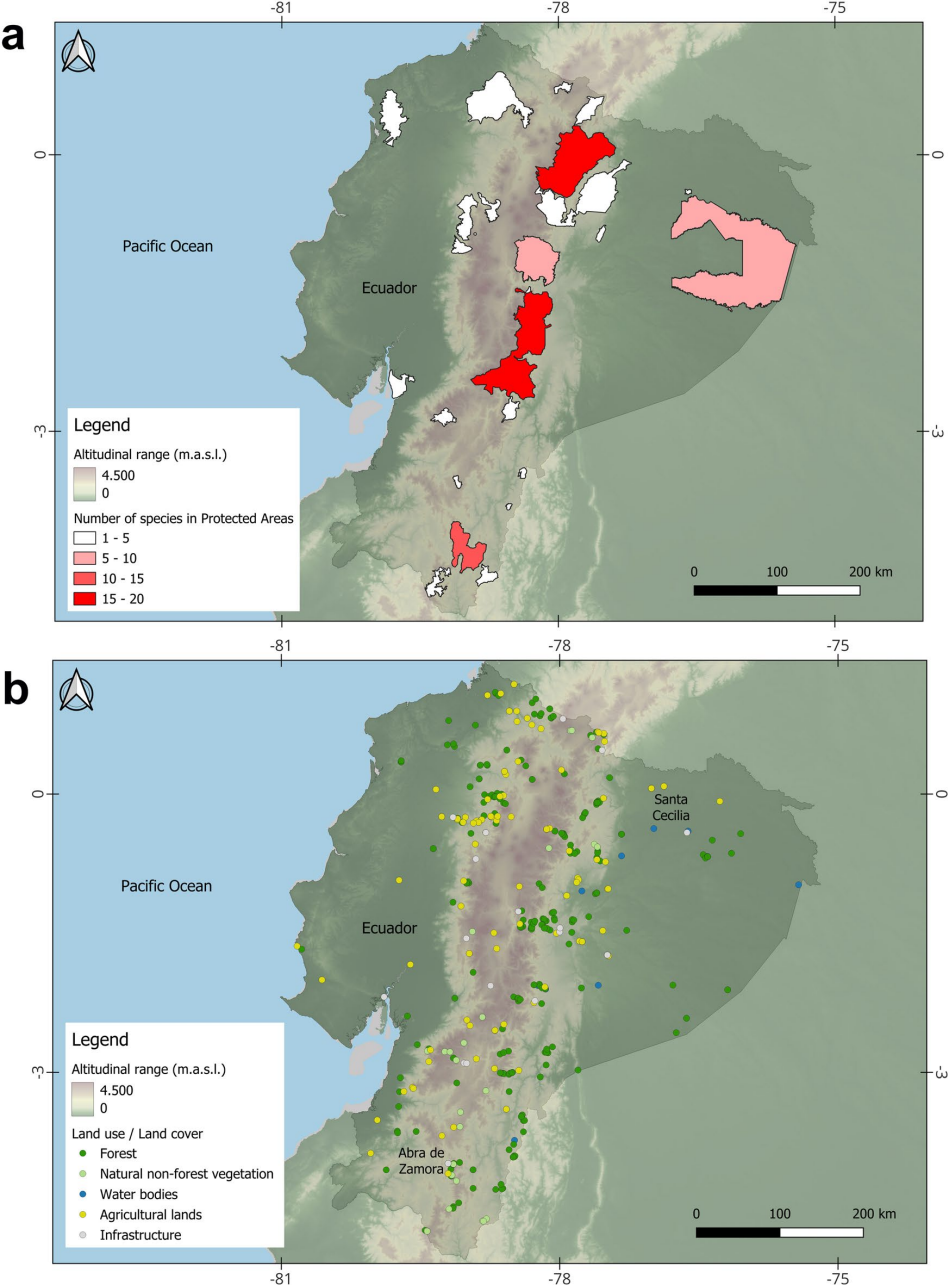
Amphibian type localities from Ecuador. (**a**) Density of type localities inside nationally protected areas from Ecuador. (**b**) Predominant type of land use/ land cover at type localities.

Of the total 505 type localities, 21.2% (107) lie inside the National Network of Protected Areas. The national parks housing the highest number of type localities are Cayambe-Coca (20 species), Sangay (16), Podocarpus (11) and Llanganates (10 species) (Fig. 4a). In terms of land cover, 26.7% (135) are in agricultural lands and 7% (36) of the type localities are in anthropic areas without vegetation (Fig. 4b).

## Patterns in Ecuadorian type locality distribution

**Fig. 5:**
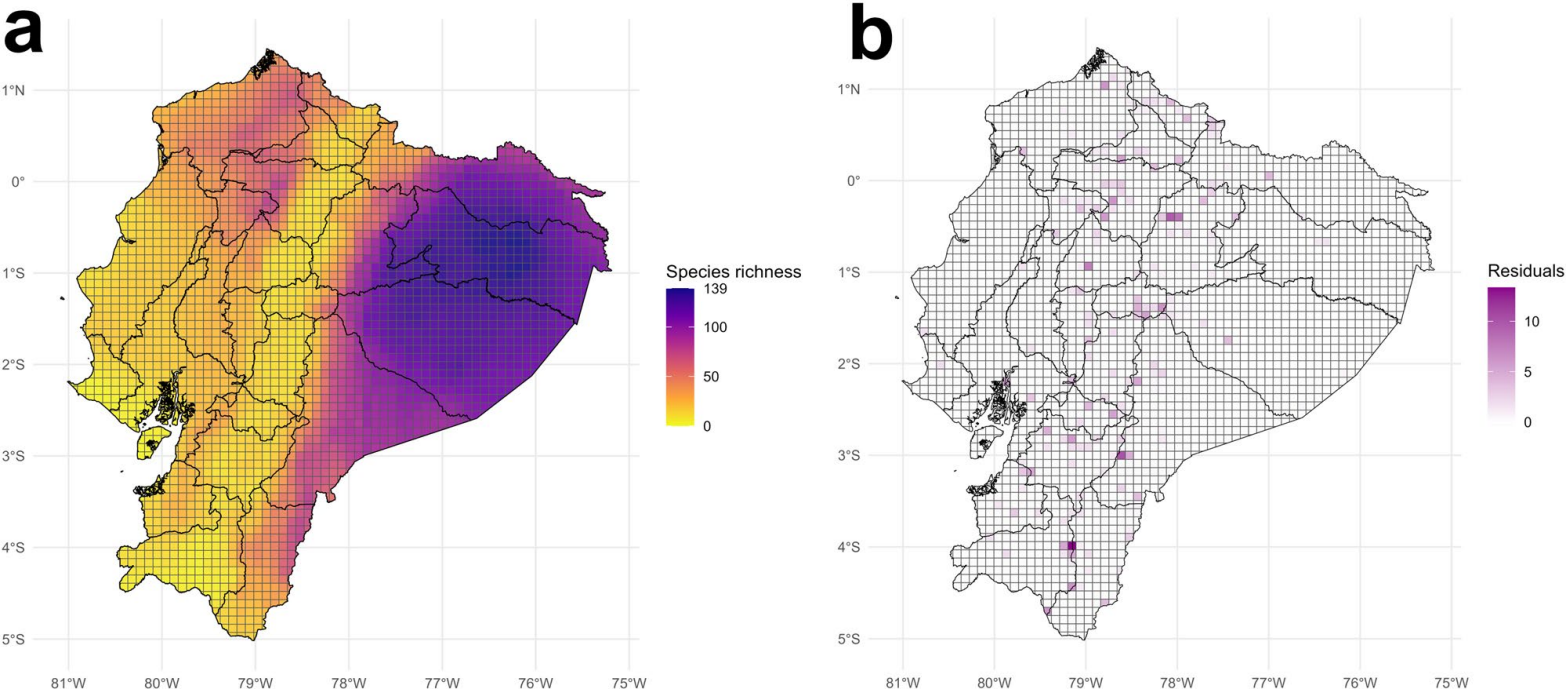
Relationship between species richness and type localities distribution. (**a**) Species richness in cells of 10 × 10 km across Ecuador. (**b**) Spatial distribution of residuals showing deviation between observed and expected counts of type localities in relation to species richness, on 10 × 10 km grid cell across Ecuador. Higher values (more intense purple color) indicate higher than expected counts of type localities in a grid cell.

Grid cells with higher species richness (Fig. 5a) had higher counts of type localities (estim. ± SE = 0.028 ± 0.004, *p* < 0.001); however, species richness increased the probability of structural zeros (estim. ± SE = 0.071 ± 0.009, *p* < 0.001). Regions such as Abra de Zamora, Tinajillas, and the road between Papallacta and Baeza showed higher than expected counts of type localities based on the model (Fig. 5b).

**Fig. 6:**
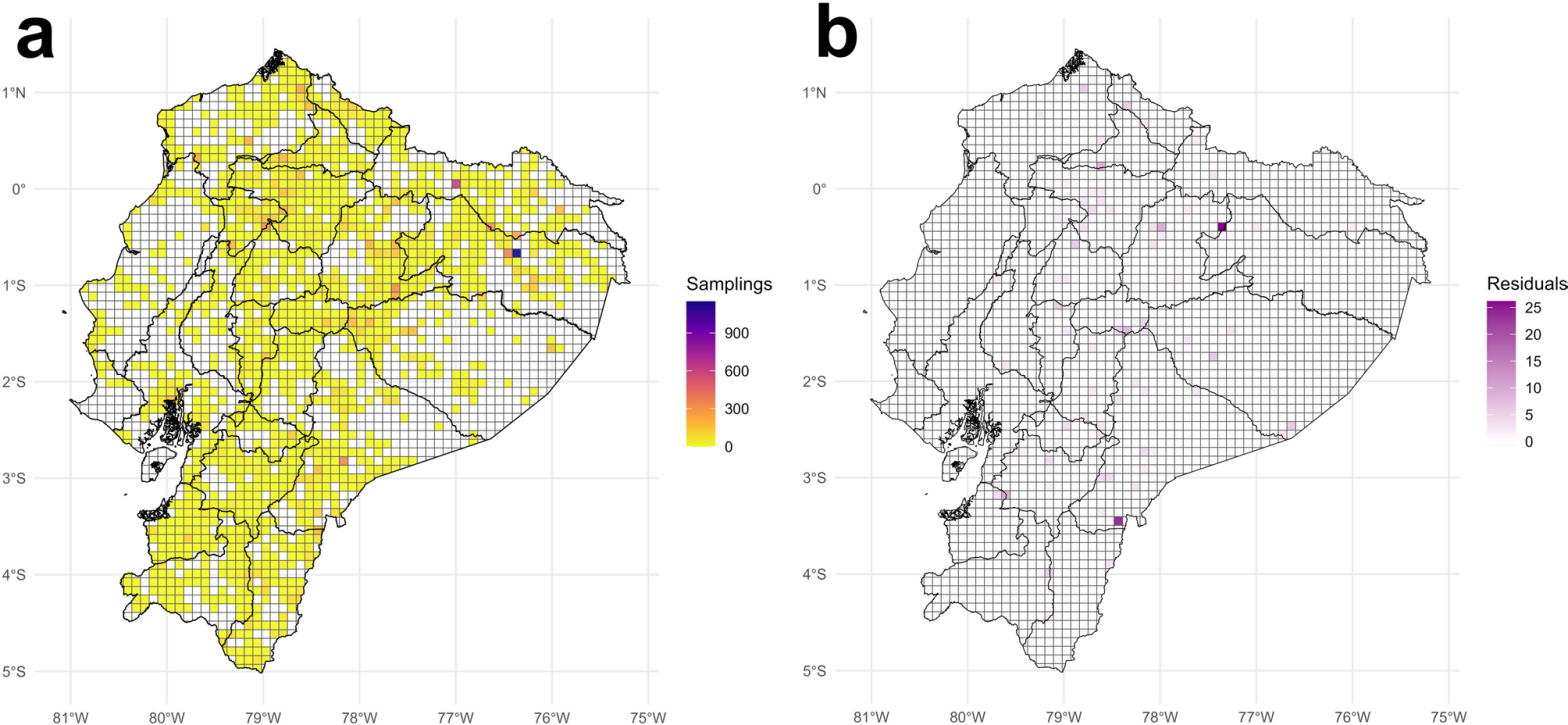
Relationship between sampling intensity and type localities distribution. (**a**) Sampling effort (number of sampling events) in cells of 10 × 10 km across Ecuador. (**b**) Spatial distribution of residuals showing deviation between observed and expected counts of type localities based on sampling effort, on 10 × 10 km grid cell across Ecuador. Higher values (more intense purple color) indicate higher than expected counts of type localities in a grid cell.

Higher sampling intensity in a grid cell (Fig. 6a) was related to a higher density of type localities (estim. ± SE = 0.0067 ± 0.001, *p* < 0.001) and a reduction in the probability of structural zero (estim. ± SE = -0.194 ± 0.026, *p* < 0.001). “San José de Moti”, the site on the lower northwestern slopes of the Sumaco Volcano, and Reserva Biológica El Quimi had higher than expected densities of type localities given their sampling intensity (Fig. 6b).

**Fig. 7:**
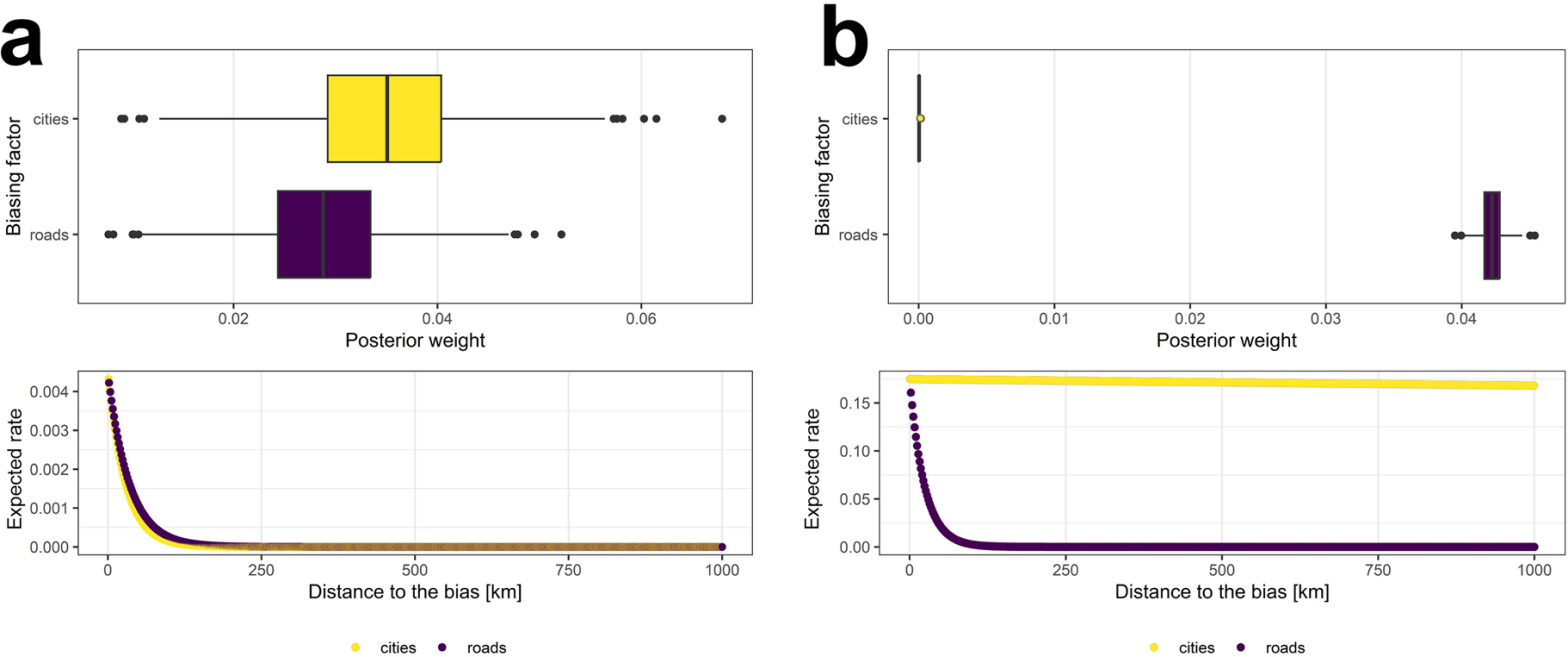
Biasing effect of accessibility factors (cities and roads) on (**a**) type localities; (**b**) sampling effort.

The accessibility factors showed a biasing effect on type localities distribution (bias weight: cities = 0.035, 95% CI 0.018–0.052; roads = 0.029, 95% CI 0.0145–0.0430; Fig. 7a), meaning that the probability of existence of a type locality in a grid cell drops with distance from both cities and roads (halved by 19.8 km distance from the roads and 24.1 km from the cities). By comparison, sampling showed a stronger and more precise effect of roads (0.04227, 95% CI  0.0406–0.0439), while the effect of cities was negligible (0.000039; Fig. 7b). Sampling probability is reduced by half at 16.4 km distance from a road.

## Discussion

Our study includes the type localities for all amphibian species reported for Ecuador to date. Although the information regarding type localities was available scattered through documents and online platforms^2^, this is the first work centralizing, georeferencing and analyzing the patterns underlying the distribution of these sites, which can be further of importance as a criterion for designating specific sites as potential natural sanctuaries^32^. Although type localities have long been recognized as important from a taxonomical point of view^33^, their conservation has rarely been proposed as a priority because their location was assumed to be the result of sampling biases and just one point location in the distribution range of the species^34^; however, their value is increased by their historical dimension and attractiveness for general public. Our results emphasize the “hotspots” of type localities, which we argue have a great value and the potential to bolster conservation actions. To our knowledge, this is the first study to explore this idea in Ecuador, and future comparisons of patterns from other taxonomic groups would improve our understanding of the factors influencing species description and conservation.

### History of amphibian species descriptions in Ecuador

The first stage (between approx. 1847–1950) was based on collections done mostly by European or North American naturalists, explorers and collectors, such as Clarence Buckley, William F. H. Rosenberg, Louis Fraser, William Clarke MacIntyre, Carl Moritz Wagner, Enrico Festa, who sent specimens to large museums, where they were described by curators, most prominently George Albert Boulenger (1858–1937), Wilhelm Peters (1815–1883), Mario Giacinto Peracca (1861–1923) or Lars Gabriel Andersson (1868–1951). The exception is represented by Marcos Jiménez de la Espada (1831–1898), who made detailed descriptions of species based on vouchers obtained by himself during his expeditions to Ecuador. During this interval, the only local collector who received recognition was the Ecuadorian Clodoveo Carrión Mora (1883–1957), who gathered several important organisms including type material for various taxa from his hometown Loja; it is however probable that many of the type specimens were in fact captured by local people. During this first stage, we see a pattern of type localities being distributed especially in the lowlands and around cities (Quito, Loja, Cuenca) in the interandean valleys. Along with geographical access and collector’s interests, older collections were probably biased towards larger, more charismatic and easier to identify species^35,36^.

The second stage (1950–2000) shows a huge influence of the Kansas University herpetology team [most distinctively William E. Duellman (1930–2022) and John D. Lynch (1942–)] that expands searches towards unexplored sites, makes intensive studies of whole amphibian communities and set up research centers. These scientists established durable collaborations and fostered the academic growth of several local graduate students that subsequently became prominent researchers leading and promoting national herpetology, paving the way for the third stage^5^. There is an increase in interest towards higher altitudes, especially Western Montane forests, with a reduction of type localities coming from the lowlands; in several instances, collections were made taking advantage of recently opened roads (e.g. Abra de Zamora) or development of oil fields (Santa Cecilia). It is during this interval that we see more local collectors receiving recognition (such as Luis A. Coloma and the Olalla family). In 1995, Luis A. Coloma publishes the first species descriptions lead by an Ecuadorian^37^.

The situation radically changes after 2000, when most of the taxonomical research is carried out by Ecuadorians. Higher proportion of type localities come from intermediate and higher altitudes, especially from the Eastern Montane Forests, which have been shown to be sites with lower alpha diversity, but with a high percentage of endemism and high allopatric speciation rates^38^. The reduction in distance between the type locality and the centroid of species distribution over time suggests the detection of species with more restricted ranges^39^, although this can also be the result of artifacts such as the refinement of species delimitation^40,41^, or lack of distribution knowledge that will be corrected in future^42^.

One limitation in our study is the quality of initial information regarding type material collection sites; for species described earlier we needed to georeference descriptive locality data, which inherently have a degree of incertitude and a possibility of error. Obscure (Americas) or erroneous (Virginia) type localities have been the source of confusion and prolonged delays in clarifying the identity of wide-spread species^43^. Collectors used to pay little attention to describing with enough detail the locality, assuming that it was well known^10^, some settlements have disappeared over time or changed their name, or the author disregarded or misspelled local toponyms^44–46^. Some researchers were able to trace such obscure locations after efforts that can only be described as detective work^45,47–49^. In cases where the location cannot be obtained, it can be beneficial for taxonomical clarity to restrict the type locality whenever possible, for example when assigning neotypes or lectotypes^50–52^.

### Geographical patterns in type localities distribution

As expected, we found that the distribution of type localities in Ecuador is partially incongruent with regions of high species richness, but rather reflects accessibility and sampling effort. Our results indicate that more intensely sampled areas are more likely to become type localities, and the number of species described from the area increases with the number of sampling occasions. On the other hand, the relationship with species richness is more complex, the model indicating that highly biodiverse cells are less likely to have a type locality, probably due to difficult access (and lack of sampling). The number of type localities in a grid cell was correlated to species richness, suggesting that for cells that are adequately sampled the number of species discovered also increases.

The Ecuadorian lowlands on both sides of the Andes show few type localities, although the reasons behind this are distinct. The western Ecuadorian lowlands have a relatively reduced species richness caused by lower precipitation, but also because of intense anthropic changes^53,54^. Additionally, both these aspects make it less attractive for researchers, further reducing the probability that species are described from these areas^55,56^. Conversely, the Ecuadorian Amazon basin is similarly underrepresented in relation to its species diversity, our results showing a surprising decrease in the probability of having a type locality in extremely diverse cells. Firstly, this is related to the lack of access to most of Amazonia, which is evident in the lack of sampling over many of the cells in this area. An additional consideration is that some of the species here have wide ranges, and their type locality lies outside Ecuador. However, it is probable that taxonomic efforts targeted towards these areas are likely to reveal endemic species, as it was suggested for other taxa^39^.

The highest number of type localities are in Montane Cloud Forest ecosystems on both sides of the Andes, with a high proportion of these species being described over the last two and a half decades. These regions are expected to hold the highest number of undescribed species at a global level^57,58^. Many such sites are logistically hard to access because of topography and climate, unsafe due to criminal organizations or military activities, and generally understudied due to limited funding and constraints, such as the difficulty of obtaining research permits; these are problems common to many hyperdiverse tropical regions^59,60^. However, we see that with the passing of time, researchers reach remote areas and reveal the presence of new, and in many cases, microendemic species.

On the other hand, long-inhabited sites hold surprises and, due to advancement in technology and more in-depth studies, new species are described even from large cities^61,62^. In cases where this is the result of taxonomical revisions of well-known species groups, the resulting species are likely to have smaller ranges and be more threatened than previously thought, with the potential of making the type localities of both the new and the original species priority sites for their conservation^63,64^.

Assuming that higher specialization and experience in particular taxa might lead to higher numbers of species descriptions, we also checked for a research bias resulting from an interest in particular families (such as harlequin frogs, glass frogs or rain frogs). However, we found that in most amphibian families occurring in Ecuador, more than half of the species were described from this country (Supplementary Fig. S1b), and we consider that this is likely related to a high endemism.

### Importance of type localities

Type specimens and their geographical site of collection (type locality) represent critical pieces of information linked to the nomenclature of a species^65^. Type locations are paramount from the systematic point of view, because additional material can be procured from the same population^66^. If the name-bearing specimen is lost, priority for the designation of new type material is given to topotypes. In cases where boundaries between species are contentious or difficult to establish, acquiring additional information such as CT-scans, DNA-grade tissue samples for molecular identification or call recordings from specimens from the type locality can be crucial for clarifying unresolved taxonomic issues and the basis for hypothesis regarding phylogenetic relationships. When multiple species share the same type locality, forming so-called “type locality hotspots”^16^, the conservation of these sites should be prioritized^67^.

Although point localities are not generally considered relevant for the distribution of a species, they are critical for microendemics (in the case of Ecuadorian amphibians, 22% of the species). Targeting areas with a high density of microendemic and/or newly described species for intense surveys is an urgent necessity, as these areas are likely to hold still unknown species^68^. Additionally, there should be an intense focus on gathering data required for robust conservation assessments, by providing basic information relevant to the Red List evaluation, such as distribution, population size and trends, habitat requirements, ongoing or potential threats^12,69–71^.

Importantly, type localities hold a historical and cultural value, since they have been visited and studied by prominent figures such as explorers, naturalists and zoologists. In many cases, these scientists made detailed descriptions of the site at the time of their expeditions, along with notes regarding the natural history and ecology of the populations, crucial for longitudinal studies of biological processes^44,72–74^. Such characteristics raise the profile of these sites, potentially making them more appealing to funding for research and conservation^75^.

### Case studies

Two grid cells are remarkable in their density of type localities, but also by having completely divergent histories and conservation pathways: Abra de Zamora and the city of Loja, and Santa Cecilia; they are addressed separately below. Beyond these, several clusters show an important mixture between cultural and biodiversity values and would benefit from improved conservation status. For example, San José de Moti has a high density of type localities of specimens collected in 1865 by the Spanish zoologist Marcos Jiménez de la Espada and currently lays outside the Ecuadorian National Network of Protected Areas. Also, the old road connecting Santo Domingo de los Colorados to Quito is a hotspot of type localities, including those related to collections made by the English explorer Edward Whymper in 1880. The area has a high concentration of threatened species^4^, as it suffers from extreme pressure from anthropic activities and lacks official protection. An impressive number of amphibian species have been described from around the road connecting the cities Baños and Puyo, both historically and during recent years; most of these type localities are found in various reserves, but the focus on the heritage importance of type localities could represent an impulse to sustainable tourism in the area.

Abra de Zamora and the city of Loja—The highest density of type localities occurs over this grid cell (14 species, of which eight are microendemics). The specimens of three species were collected inside the “hoya de Loja”, an interandean valley that houses the city of Loja (approx. 2000 m a.s.l.); the collections were made by the emblematic Ecuadorian naturalist Clodoveo Carrión during the 1930s. Due to urbanization, these species have all experienced reductions in their populations and have not been encountered in the city for decades. The rest of the type localities lie close to the old road connecting Loja and Zamora cities, on the crest of the Cordillera de los Andes (approx. 2800 m a.s.l.) going down the Amazonian slope. They are based on collections made by the team of herpetologists from the Kansas University (mainly Duellman and Lynch) during three visits between 1968 and 1975, but also by the Universidad Técnica Particular de Loja (UTPL) in 2018. Abra de Zamora has been recognized as a hotspot of diversity; even if the alpha diversity at the site is smaller compared to the lowlands, it is remarkable for its high proportion of microendemic species, representing a center of amphibian diversification^76^. The habitat is a mixture of evergreen upper montane forest and subpáramo or evergreen elfin forest. Despite being close to the city, this site is relatively well preserved, maintaining its natural vegetation cover, the main threats being represented by cattle farming, chytrid fungus and the introduction of exotic species (Rainbow Trout *Oncorhynchus mykiss* and Bullfrog *Aquarana catesbeiana*)^44^; we can assume that climate change will also have a negative impact on these high-altitude species^77,78^. Over the last several years, the efforts of the NGO Naturaleza y Cultura Internacional have been focused towards extending the Podocarpus National Park to include Abra de Zamora, which would improve the conservation status of this important biodiversity hotspot. Also, together with the UTPL, they carried out several awareness campaigns for Loja residents, promoting a sense of local pride and responsibility towards this unique fauna that can be found less than 15 km away from the city.

Santa Cecilia—This is the second densest accumulation of type localities, represented by eleven species, all wide-ranging. The amphibian types from Santa Cecilia (ten holotypes and a neotype) were collected by the Kansas University team between 1968 and 1975. This is a swampy Amazonian rainforest, at an elevation of 340 m a.s.l., on the banks of the Aguarico River. Upon his first fieldtrip in 1966, Duellman described the site as “virgin or only slightly disturbed tropical forest”, near what was then a small indigenous settlement (Santa Cecilia) and the newly established Texaco oil camp, and next to an airstrip which provided the connection to the rest of Ecuador^79^. Duellman remarks that the alpha diversity of herpetofauna was one of the highest in the world; it is also one of the best documented, the team collecting from this small area 7765 specimens of amphibians and reptiles in less than 10 years^80^. However, the situation at the site soon changed. In 1971, the road from Quito was built (now the Panamericana). By 1993, Marty Crump, part of the original KU team that spent over a year documenting the ecology and reproductive strategies of the anuran community in Santa Cecilia, was appalled by the spread of the colonization and radical anthropical modifications that affected the site^81^. Currently, the biodiversity at Santa Cecilia has been severely impacted by oil exploitation, including oil runoff and spills^82^, but also by deforestation^83^ and expansion of the army camp and agriculture, all leading to drastic changes in the amphibian community^81^.

### Conservation gaps and opportunities

Amphibians are experiencing a decline at the global level, and this is exacerbated in Ecuador, where 57% are classified as threatened^4^. For the country, the change in land use conversion is dramatic^53,84^. We found that 34% of the Ecuadorian amphibian type localities are in areas experiencing drastic change in land-use, which is one of the main source of species decline^85,86^. While type localities that are closer to inhabited centers and roads are mostly impacted by extensive deforestation, agriculture and urbanization, the ones that are in remote areas frequently face destruction due to mining activities (oil, gold, copper)^87^. Furthermore, there are more pervasive but still damaging impacts such as disease or invasive species and climate change, which can impact even relatively unmodified natural ecosystems^88,89^. Especially microendemics species, which have highly specialized habitat requirements and narrow distribution ranges, can face extinction in the case of changes close to the type localities. For the type localities situated in the highlands, in addition to land-use changes, it is expected that the ecosystems will be disproportionately affected by climate change, which could lead to translocation of type populations^90^. This should be the focus of targeted monitoring, and generate adequate protection measures.

Our results indicate that only 21% of the Ecuadorian type localities are inside nationally protected areas. Minimally modified borders of the existing national parks or reserves would improve the protected status of some of these sites (e.g., Abra de Zamora, San José de Moti). However, a “paper expansion” of the existing protected area network, which is already understaffed, underfunded and lacks the capacity to tackle external pressures, is not a remedy for all. Very small reserves are particularly vulnerable to edge effects, which can compromise their capacity to sustain viable amphibian populations over time^91^. For this reason, conservation efforts should not rely solely on defining minimum reserve sizes, but rather on situating these areas within a broader, connected landscape. In this context, connectivity becomes more effective when conservation is supported by diverse governance systems, including Indigenous and community-managed lands that often retain relatively intact ecological conditions. Strengthening links among these areas through ecological corridors and coordinated management can facilitate dispersal, maintain gene flow, and allow species to respond more effectively to ongoing environmental change^92^.

The very reasons that might make a site more likely to become a type locality – the ease of access, closeness to inhabited centers – are what makes it more vulnerable to habitat decline, but also likely candidates for successful fundraising or environmental education campaigns. They can have a higher intrinsic appeal to the public in general, due to a bias in human interest^93^. Although science should view site conservation using impersonal criteria, pragmatism dictates that type localities can have more traction. Type localities can become educational landmarks, fostering pride and awareness in the local community. In the case of species that bear the names of local places or indigenous people, they can connect people to natural heritage, sparking interest and engagement in conservation. We hope our findings will promote further research into other taxa to reveal hotspots of type localities and their particularities, supporting integrative conservation actions.

## Supplementary Information

Below is the link to the electronic supplementary material.

Supplementary Material 1

Supplementary Material 2

## Data Availability

The dataset generated for this work is available as Supplementary Material, Table S1.

## References

[1] Yang, R. et al. Cost-effective priorities for the expansion of global terrestrial protected areas: Setting post-2020 global and national targets. *Sci. Adv.* **6**, eabc3436. 10.1126/sciadv.abc3436 (2020). 32917690 10.1126/sciadv.abc3436 PMC11206530

[2] Frost, D. R. Amphibian Species of the World: An Online Reference (2025). https://amphibiansoftheworld.amnh.org/index.php.

[3] Tapley, B. et al. The disparity between species description and conservation assessment: A case study in taxa with high rates of species discovery. *Biol. Conserv.* **220**, 209–214 (2018).

[4] Ortega-Andrade, H. M. et al. Red List assessment of amphibian species of Ecuador: a multidimensional approach for their conservation. *PLOS One*. **16**, e0251027. 10.1371/journal.pone.025102 (2021). 33956885 10.1371/journal.pone.0251027 PMC8101765

[5] Coloma, L. A. & Duellman, W. E. *An Introduction to the Amphibians of Ecuador: Diversity, Conservation, and Cultural History* (CRC, 2024).

[6] Rivera Correa, M. et al. Amphibians in Zootaxa: 20 years documenting the global diversity of frogs, salamanders, and caecilians. *Zootaxa* **4979**, 57–69 (2021). 10.11646/zootaxa.4979.1.9 34187014

[7] Liu, J., Slik, F., Zheng, S. & Lindenmayer, D. B. Undescribed species have higher extinction risk than known species. *Conserv. Lett.* **15**, e12876. 10.1111/conl.12876 (2022).

[8] Daru, B. H. & Rodriguez, J. Mass production of unvouchered records fails to represent global biodiversity patterns. *Nat. Ecol. Evol.* **7**, 816–831 (2023). 37127769 10.1038/s41559-023-02047-3

[9] Dunn, E. & Stuart, L. On the legality of restriction of type locality. *Science* **113**, 677–678 (1951). 14845713 10.1126/science.113.2946.677

[10] Moreau, K., Hopkins, G. & Hayman, R. The type-localities of some African Mammals. *Proc. Zool. Soc. Lond.* **115**, 387–447 (1946).

[11] Donoso, D. A., Salazar, F., Maza, F., Cárdenas, R. E. & Dangles, O. Diversity and distribution of type specimens deposited in the invertebrate section of the Museum of Zoology QCAZ, Quito, Ecuador. *Ann. de la. Société Entomologique de France*. **45**, 437–454 (2009).

[12] Araujo, H. F., Machado, C. C. & da Silva, J. M. C. The distribution and conservation of areas with microendemic species in a biodiversity hotspot: a multi-taxa approach. *PeerJ* **12**, e16779. 10.7717/peerj.16779 (2024). 38239293 10.7717/peerj.16779 PMC10795537

[13] Brundu, G. et al. At the intersection of cultural and natural heritage: Distribution and conservation of the type localities of Italian endemic vascular plants. *Biol. Conserv.* **214**, 109–118 (2017).

[14] Erwin, T. L. An evolutionary basis for conservation strategies. *Science* **253**, 750–752 (1991). 17835489 10.1126/science.253.5021.750

[15] Plumptre, A. J. et al. Targeting site conservation to increase the effectiveness of new global biodiversity targets. *One Earth*. **7**, 11–17 (2024).

[16] Azevedo-Santos, V. M. & Ottoni, F. P. Conserving the type locality hotspots. *Bioscience* **75**, 609–611 (2025).

[17] Ron, S. R., Merino-Viteri, A. & Ortiz, D. A. Anfibios del Ecuador. Version 2024.0 (2024). https://bioweb.bio/faunaweb/amphibiaweb.

[18] Böhme, W. Presence of *Agama weidholzi* Wettstein, 1932 in The Gambia, West Africa. *Salamandra* **41**, 155–158 (2005).

[19] Ortega-Andrade, H. M. et al. Insights from integrative systematics reveal cryptic diversity in *Pristimantis* frogs (Anura: Craugastoridae) from the Upper Amazon Basin. *PLOS One*. **10**, e0143392. 10.1371/journal.pone.0143392 (2015). 26600198 10.1371/journal.pone.0143392 PMC4658055

[20] Castillo-Urbina, E., Vences, M., Aguilar-Puntriano, C., Glaw, F. & Köhler, J. Contributing to the taxonomic inventory of green-colored rain frogs: A new species of the *Pristimantis lacrimosus* group (Anura: Strabomantidae) from the southern Cordillera Azul, central Peru. *Vertebr Zool.* **73**, 1047–1061 (2023).

[21] Cisneros-Heredia, D. F. The hitchhiker wave: non-native small terrestrial vertebrates in the Galápagos. in *Understanding Invasive Species in the Galapagos Islands - From the Molecular to the Landscape* (eds Torres, M. L .& Mena, C. F.) 95–139 (2018).

[22] R Core Team. *R: A language and environment for statistical computing* (R Foundation for Statistical Computing, 2025).

[23] Tennekes, M. & tmap Thematic Maps in R. *J. Stat. Softw.* **84**, 1–39 (2018).30450020

[24] Ron, S. R., Guayasamin, J. M. & Menéndez-Guerrero, P. Biodiversity and conservation status of Ecuadorian amphibians. in *Amphibian Biology 9. Status of Decline of Amphibians: Western Hemisphere Part 2: Brazil, Uruguay, Columbia, and Ecuador* (eds. Heatwole, H., Barrio-Amorós, C. L. & Wilkinson, J. W.) 129–170 (Surrey Beatty & Sons, 2011).

[25] Holguín, W., Aguilar, C., Terán, K., Rodríguez, A. & Josse, C. *Documento de Bases Teóricas de Algoritmo (ATBD) MapBiomas Ecuador Colección 2.0, Apéndice Ecuador - Colección 2.0 de Mapas Anuales de Cobertura y Uso del Suelo de la Amazonía*. (Fundación de Estudios Ecológicos Ecociencia, 2024).

[26] IUCN. The IUCN Red List of Threatened Species (2025). https://www.iucnredlist.org.

[27] Joppa, L. N. et al. Impact of alternative metrics on estimates of extent of occurrence for extinction risk assessment. *Conserv. Biol.* **30**, 362–370 (2016). 26183938 10.1111/cobi.12591

[28] Diniz-Filho, J. A. F. et al. Macroecological correlates and spatial patterns of anuran description dates in the Brazilian Cerrado. *Global Ecol. Biogeogr.* **14**, 469–477 (2005).

[29] Zeileis, A., Kleiber, C. & Jackman, S. Regression models for count data in R. *J. Stat. Softw.* **27**, 1–25 (2008).

[30] Engemann, K. et al. Limited sampling hampers big data estimation of species richness in a tropical biodiversity hotspot. *Ecol. Evol.* **5**, 807–820 (2015). 25692000 10.1002/ece3.1405 PMC4328781

[31] Zizka, A., Antonelli, A. & Silvestro, D. sampbias, a method for quantifying geographic sampling biases in species distribution data. *Ecography* **44**, 25–32 (2021).

[32] Stuckey, R. L. & Pringle, J. S. Type localities of vascular plants first described from Ohio. *SIDA Contrib. Bot.* **20**, 1677–1692 (2003).

[33] Bailey, V. The importance of types and type localities. *J. Mammal*. **14**, 241–243 (1933).

[34] Dennis, R. & Thomas, C. Bias in butterfly distribution maps: the influence of hot spots and recorder’s home range. *J. Insect Conserv.* **4**, 73–77 (2000).

[35] Costello, M. J., Lane, M., Wilson, S. & Houlding, B. Factors influencing when species are first named and estimating global species richness. *Glob Ecol. Conserv.* **4**, 243–254 (2015).

[36] Guedes, J. J. M., Moura, M. R., Alexandre, F. & Diniz-Filho, J. Species out of sight: elucidating the determinants of research effort in global reptiles. *Ecography* **2023**, e06491. 10.1111/ecog.06491 (2023).

[37] Coloma, L. A. Ecuadorian frogs of the genus *Colostethus* (Anura: Dendrobatidae). *Misc Publ Univ. Kans.* **87**, 1–72 (1995).

[38] Tobar-Suárez, C., Urbina-Cardona, N., Villalobos, F. & Pineda, E. Amphibian species richness and endemism in tropical montane cloud forests across the Neotropics. *Biodivers. Conserv.* **31**, 295–313 (2022).

[39] Frateles, L. E. F., da Silva Jr, N. J., Terribile, L. C. & Diniz-Filho, J. A. F. Linnean shortfall and space‐time patterns in species description of New World coralsnakes (Serpentes: Elapidae). *Zool. Scr.* **53**, 299–311 (2024).

[40] dos Santos, S. P., Ibáñez, R. & Ron, S. R. Systematics of the *Rhinella margaritifera* complex (Anura, Bufonidae) from western Ecuador and Panama with insights in the biogeography of *Rhinella alata*. *ZooKeys* 109–145 (2015). 10.3897/zookeys.501.8604 PMC4432321 25987881

[41] Varela-Jaramillo, A., Streicher, J. W., Venegas, P. J. & Ron, S. R. Three new species of torrent treefrogs (Anura, Hylidae) of the *Hyloscirtus bogotensis* group from the eastern Andean slopes and the biogeographic history of the genus. *ZooKeys* **1231**, 233–292 (2025). 40124314 10.3897/zookeys.1231.124926 PMC11926613

[42] Meiri, S. et al. Extinct, obscure or imaginary: the lizard species with the smallest ranges. *Divers. Distrib.* **24**, 262–273 (2018).

[43] Lötters, S. et al. A roadmap for harlequin frog systematics, with a partial revision of Amazonian species related to *Atelopus spumarius*. *Zootaxa* **5571**, 1–76 (2025). 40173732 10.11646/zootaxa.5571.1.1

[44] Székely, P., Eguiguren, J. S., Ordóñez-Delgado, L., Armijos-Ojeda, D. & Székely, D. Fifty years after: a taxonomic revision of the amphibian species from the Ecuadorian biodiversity hotspot Abra de Zamora, with description of two new *Pristimantis* species. *PLOS One*. **15**, e0238306. 10.1371/journal.pone.0238306 (2020). 32911497 10.1371/journal.pone.0238306 PMC7482940

[45] González-Fernández, J. E. Anfibios colectados por la Comisión Científica del Pacífico (entre 1862 y 1865) conservados en el Museo Nacional de Ciencias Naturales de Madrid. *Graellsia* **62**, 111–158 (2006).

[46] Peters, J. A. The frog genus *Atelopus* in Ecuador (Anura: Bufonidae). *Smithson. Contrib. Zool.* **145**, 1–49 (1973).

[47] Cisneros-Heredia, D. F. The type localities of *Anolis aequatorialis* Werner, 1894 (Sauria: Iguania: Dactyloidae) and *Pristimantis appendiculatus* (Werner, 1894) (Amphibia: Anura: Craugastoridae). *Zootaxa* **4216**, 190–196 (2017). 10.11646/zootaxa.4216.2.5 28183129

[48] Cisneros-Heredia, D. F. Restriction of the type locality Los Puentes, Ecuador, for several species of Arachnida, Gastropoda and plants. *Zootaxa* **5642**, 389–394 (2025). 41119099 10.11646/zootaxa.5642.4.6

[49] Gardner, A. L. *Proechimys semispinosus* (Rodentia: Echimyidae): Distribution, type locality, and taxonomic history. *Proc. Biol. Soc. Wash.* **96**, 134–144 (1983).

[50] Braby, M. F., Hsu, Y. F. & Lamas, G. How to describe a new species in zoology and avoid mistakes. *Zool. J. Linn. Soc.* **202**, zlae043 (2024).

[51] Heyer, W. R. Variation and taxonomic clarification of the large species of the *Leptodactylus pentadactylus* species group (Amphibia: Leptodactylidae) from Middle America, northern South America, and Amazonia. *Arq. Zool. Sao Paulo*. **37**, 269–348 (2005).

[52] Duellman, W. E. & Wiens, J. J. Hylid frogs of the genus *Scinax* Wagler, 1830, in Amazonian Ecuador and Peru. *Occas Pap Mus. Nat. Hist. Univ. Kans.* **153**, 1–57 (1993).

[53] Ochoa-Brito, J. I., Ghosh, A. & Hijmans, R. J. Cropland expansion in Ecuador between 2000 and 2016. *PLOS One*. **18**, e0291753. 10.1371/journal.pone.0291753 (2023). 37725616 10.1371/journal.pone.0291753 PMC10508625

[54] Rivas, C. A., Guerrero-Casado, J. & Navarro-Cerrillo, R. M. Functional connectivity across dominant forest ecosystems in Ecuador: A major challenge for a country with a high deforestation rate. *J. Nat. Conserv.* **78**, 126549. 10.1016/j.jnc.2023.126549 (2024).

[55] Oliveira, U. et al. The strong influence of collection bias on biodiversity knowledge shortfalls of Brazilian terrestrial biodiversity. *Divers. Distrib.* **22**, 1232–1244 (2016).

[56] Sastre, P. & Lobo, J. M. Taxonomist survey biases and the unveiling of biodiversity patterns. *Biol. Conserv.* **142**, 462–467 (2009).

[57] Vieites, D. R. et al. Vast underestimation of Madagascar’s biodiversity evidenced by an integrative amphibian inventory. *PNAS* **106**, 8267–8272 (2009). 19416818 10.1073/pnas.0810821106 PMC2688882

[58] Moura, M. R. & Jetz, W. Shortfalls and opportunities in terrestrial vertebrate species discovery. *Nat. Ecol. Evol.* **5**, 631–639 (2021). 33753900 10.1038/s41559-021-01411-5

[59] Saboyá Acosta, L. P. & Urbina-Cardona, J. N. Current state of knowledge of páramo amphibians in Colombia: Spatio temporal trends and information gaps to be strengthened for effective conservation. *Trop. Conserv. Sci.* **16**. 10.1177/19400829231169984 (2023).

[60] Carné, A. et al. Microendemism in Madagascar: small ranges or sampling gaps? The case of the frog *Wakea madinika*. *Salamandra* **61**, 160–170 (2025).

[61] Székely, P., Székely, D., Ordóñez-Delgado, L., Armijos-Ojeda, D. & Vörös, J. Our unknown neighbor: A new species of rain frog of the genus *Pristimantis* (Amphibia: Anura: Strabomantidae) from the city of Loja, southern Ecuador. *PLOS One*. **16**, e0258454. 10.1371/journal.pone.0258454 (2021). 34705824 10.1371/journal.pone.0258454 PMC8550592

[62] Carvajal-Endara, S. et al. Phylogenetic systematics, ecology, and conservation of marsupial frogs (Anura: Hemiphractidae) from the Andes of southern Ecuador, with descriptions of four new biphasic species. *Zootaxa* **4562**, 1–102 (2019). 10.11646/zootaxa.4562.1.1 31716564

[63] Páez, N. B. & Ron, S. R. Systematics of *Huicundomantis*, a new subgenus of *Pristimantis* (Anura, Strabomantidae) with extraordinary cryptic diversity and eleven new species. *ZooKeys* **868**, 1–112 (2019). 31406482 10.3897/zookeys.868.26766 PMC6687670

[64] Zumel, D., Buckley, D. & Ron, S. R. The *Pristimantis trachyblepharis* species group, a clade of miniaturized frogs: description of four new species and insights into the evolution of body size in the genus. *Zool. J. Linn. Soc.* **195**, 315–354 (2022).

[65] Mayr, E. *Principles of Systematic Zoology* (McGraw-Hill Book Company, 1969).

[66] Bell, R. C. et al. The type locality project: Collecting genomic-quality, topotypic vouchers and training the next generation of specimen-based researchers. *Syst. Biodivers.* **18**, 557–572 (2020).

[67] Watt, J. C. Conservation and type localities of New Zealand Coleoptera, and notes on collectors 1770–1920. *J. R. Soc. N. Z.* **7**, 79–91 (1977).

[68] Rheindt, F. E. et al. A lost world in Wallacea: Description of a montane archipelagic avifauna. *Science* **367**, 167–170 (2020). 31919216 10.1126/science.aax2146

[69] Ponder, W. F., Carter, G., Flemons, P. & Chapman, R. Evaluation of museum collection data for use in biodiversity assessment. *Conserv. Biol.* **15**, 648–657 (2001).

[70] Dubois, A. The relationships between taxonomy and conservation biology in the century of extinctions. *C R Biol.* **326**, 9–21 (2003). 14558444 10.1016/s1631-0691(03)00022-2

[71] Burlakova, L. E. et al. Endemic species: contribution to community uniqueness, effect of habitat alteration, and conservation priorities. *Biol. Conserv.* **144**, 155–165 (2011).

[72] de la Jiménez, M. *Vertebrados del viaje al Pacífico verificado de 1862 a 1865 por una comisión de naturalistas enviada por el Gobierno Español. Batracios* (A. Miguel Ginesta, 1875).

[73] Sklenář, P. et al. Distribution changes in páramo plants from the equatorial high Andes in response to increasing temperature and humidity variation since 1880. *Alp. Bot.* **131**, 201–212 (2021).

[74] Duellman, W. E. & Crump, M. Speciation in frogs of the *Hyla parviceps* group in the upper Amazon basin. *Occas Pap Mus. Nat. Hist. Univ. Kans.* **23**, 1–40 (1974).

[75] Guénard, B. et al. Limited and biased global conservation funding means most threatened species remain unsupported. *PNAS* **122**, e2412479122. 10.1073/pnas.2412479122 (2025). 39993186 10.1073/pnas.2412479122 PMC11892620

[76] Duellman, W. E. *The South American Herpetofauna: Its Origin, Evolution, and Dispersal* Vol. 7 (Museum of Natural History, University of Kansas, 1979).

[77] Alves-Ferreira, G. et al. Climate change is projected to shrink phylogenetic endemism of Neotropical frogs. *Nat. Commun.* **16**, 3713. 10.1038/s41467-025-59036-2 (2025). 40251164 10.1038/s41467-025-59036-2 PMC12008241

[78] Menéndez-Guerrero, P. A., Green, D. M. & Davies, T. J. Climate change and the future restructuring of Neotropical anuran biodiversity. *Ecography* **43**, 222–235 (2020).

[79] Duellman, W. E. *Herpetology at Kansas: A Centennial History* (Society for the Study of Amphibians and Reptiles, 2015).

[80] Duellman, W. E. *The Biology of an Equatorial Herpetofauna in Amazonian Ecuador* Vol. 65 (University of Kansas Lawrence, 1978).

[81] Crump, M. L. *In search of the golden frog* (University of Chicago Press, 2000).

[82] Zambrano Soledispa, A. L. *Pueblos Indigenas vs. Texaco (Chevron): un análisis de caso del derramamiento de petróleo en la Amazonia Ecuatorian* (Universidad Federal de la Integración Latino- Americana, 2019).

[83] Guartatanga Pilatasig, J. E. *Evaluación del cambio climático por efecto de la deforestación a los bosques nativos en la parroquia Santa Cecilia, cantón Lago Agrio, provincia de Sucumbíos, Ecuador* (Universidad Técnica de Cotopaxi, 2025).

[84] Tapia-Armijos, M. F., Homeier, J., Espinosa, C. I., Leuschner, C. & De La Cruz, M. Deforestation and forest fragmentation in South Ecuador since the 1970s–losing a hotspot of biodiversity. *PLOS One*. **10**, e0133701. 10.1371/journal.pone.0133701 (2015). 26332681 10.1371/journal.pone.0133701 PMC4557835

[85] Newbold, T. Future effects of climate and land-use change on terrestrial vertebrate community diversity under different scenarios. *Proc. R Soc. Lond. B Biol. Sci.* **285**, 20180792. 10.1098/rspb.2018.0792 (2018). 10.1098/rspb.2018.0792 PMC6030534 29925617

[86] Newbold, T. et al. Global effects of land use on local terrestrial biodiversity. *Nature* **520**, 45–50 (2015). 25832402 10.1038/nature14324

[87] Roy, B. A. et al. New mining concessions could severely decrease biodiversity and ecosystem services in Ecuador. *Trop. Conserv. Sci.* **11**, 1940082918780427. 10.1177/1940082918780427 (2018).

[88] Martín-Torrijos, L. et al. Rainbow trout (*Oncorhynchus mykiss*) threaten Andean amphibians. *Neotrop. Biodivers.* **2**, 26–36 (2016).

[89] Menéndez-Guerrero, P. A., Davies, T. J. & Green, D. M. Extinctions of threatened frogs may impact ecosystems in a global hotspot of anuran diversity. *Herpetologica* **76**, 121–131 (2020).

[90] Fajardo, J. et al. The performance of protected-area expansions in representing tropical Andean species: Past trends and climate change prospects. *Sci. Rep.* **13**, 966. 10.1038/s41598-022-27365-7 (2023). 36653418 10.1038/s41598-022-27365-7 PMC9849396

[91] Ríos-Alvear, G. et al. Key connectivity areas in the Llanganates-Sangay ecological corridor in Ecuador: A participative multicriteria analysis based on a landscape species. *Landsc. Urban Plan.* **246**, 105039. 10.1016/j.landurbplan.2024.105039 (2024).

[92] Duarte Ritter, C. et al. Indigenous territories and protected areas are crucial for ecosystem connectivity in the Amazon basin. *PNAS* **122**, e2418189122. 10.1073/pnas.2418189122 (2025). 40720645 10.1073/pnas.2418189122 PMC12337320

[93] Veríssimo, D. et al. Increased conservation marketing effort has major fundraising benefits for even the least popular species. *Biol. Conserv.* **211**, 95–101 (2017).

