# Supplementary material for: Patterns in the distribution of Ecuadorian amphibian type localities
Source: Sci Rep. 2026 May 14;16:21991. doi: 10.1038/s41598-026-53084-4 (PMC13365574; doi:10.1038/s41598-026-53084-4)
Supplement: Supplementary file 2 — Supplementary Material 2 [file 41598_2026_53084_MOESM2_ESM.pdf]

Supplementary Information

Patterns in the distribution of Ecuadorian amphibian type localities

Diana Székely[1,2,*], Diego Armijos-Ojeda[1,3,4], Paul Székely[1,2,3]

[1] Museo de Zoología, Universidad Técnica Particular de Loja, San Cayetano Alto, calle París s/n, Loja, 110107, Ecuador

[2] Faculty of Natural and Agricultural Sciences, Ovidius University Constanța, Al. Universității no.1, Constanța 900470, Romania

[3] Laboratorio de Ecología Tropical y Servicios Ecosistémicos (EcoSs-Lab), Facultad de Ciencias Exactas y Naturales, Departamento de Ciencias Biológicas y Agropecuarias, Universidad Técnica Particular de Loja, San Cayetano Alto s/n, Loja, 110107, Ecuador

[4] Instituto Nacional de Biodiversidad INABIO, Rumipamba no. 341, Quito 170135, Ecuador

*Corresponding author: Diana Székely; email: dszekely@utpl.edu.ec

Scientific Reports

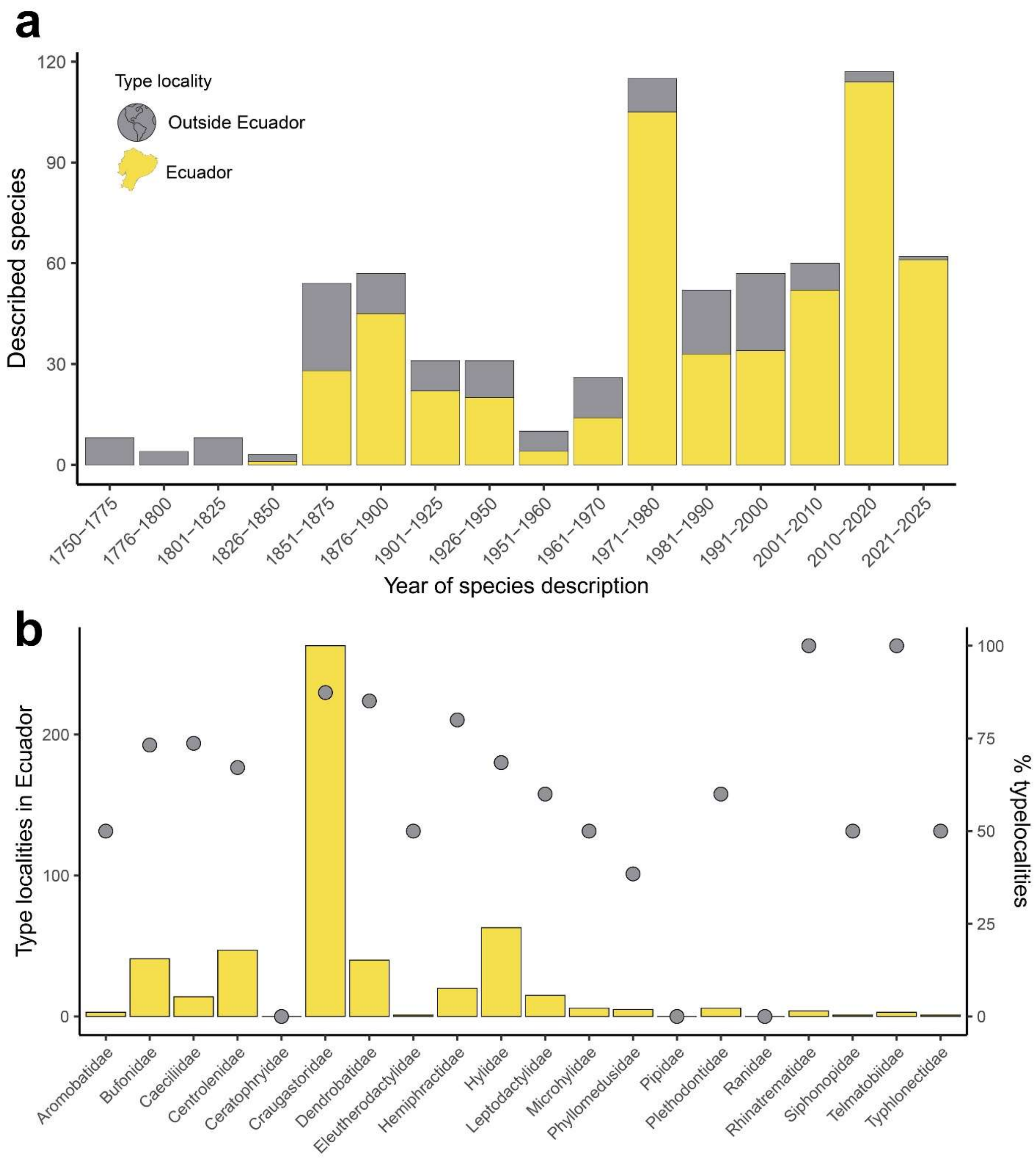

Supplementary Fig. 1. a. Location of the type localities for the 695 amphibian species which have been reported from Ecuador, according to the time interval when they were described. b. Amphibian species that have their type localities located in Ecuador, according to the family, in absolute numbers (yellow columns, left y-axis) and percentage out of the total reported from Ecuador (grey dots, right y-axis).
